# Hydrogen at symmetric tilt grain boundaries in aluminum: segregation energies and structural features

**DOI:** 10.1038/s41598-022-23535-9

**Published:** 2022-11-18

**Authors:** Cláudio M. Lousada, Pavel A. Korzhavyi

**Affiliations:** Department of Materials Science and Engineering, KTH Royal Institute of Technology, 100 44 Stockholm, Sweden

**Keywords:** Energy, Materials for energy and catalysis, Structure of solids and liquids

## Abstract

Aluminum is envisioned to be an important material in future hydrogen-based energy systems. Here we report an ab initio investigation on the interactions between H-atoms and common grain boundaries (GBs) of fcc Al: Σ9, Σ5, Σ11 and Σ3. We found that upon segregation to the GBs, single H-atoms can cause displacement of Al-atoms. Increasing their concentration revealed large cooperative effects between H-atoms that favor the segregation when other H-atoms are bound at neighboring sites. This makes these GBs able to accommodate high concentrations of H-atoms with considerable segregation energies per atom. Structural analyses derived from Laguerre–Voronoi tessellations show that these GBs have many interstitial sites with higher symmetry than the bulk tetrahedral interstitial site. Many of those sites have also large volumes and higher coordination numbers than the bulk sites. These factors are the increased driving force for H-atom segregation at the studied GBs in Al when compared to other metals. These GBs can accommodate a higher concentration of H-atoms which indicates a likely uniform distribution of H-atoms at GBs in the real material. This suggests that attempting to mitigate hydrogen uptake solely by controlling the occurrence of certain GBs may not be the most efficient strategy for Al.

## Introduction

In the transition to a society where hydrogen is widely used as fuel, aluminum is a material with an important role as a component of vessels for hydrogen storage and transportation^1–4^ and as a structural component of fuel cells^5,6^. Some studies propose Al and its alloys as a means to produce H_2_(g) from water^7^. In hydrogen energy systems Al is envisioned to be a component of hydrogen storage tanks not only for mobile applications but also for industrial applications where hydrogen will replace fossil-based fuels or chemicals such as coke in steel making^8^. In the hydrogen transportation topic, the European Union is planning to gradually blend H_2_(g) into the natural gas distribution network, starting from a small fraction of a few % of hydrogen until the network of pipelines is fully repurposed for H_2_(g) transport^9–11^. Because Al is used in parts of pipeline systems, the compatibility between Al and H_2_(g) is relevant for this planned transition.

H-atoms in the lattice of metals can affect diverse physical–chemical properties of the materials often with considerable effects in their performance. In the first stages of the process of absorption of H-atoms by a solid, H_2_(g) must dissociate at the surface of the material or alternatively, other chemical reactions must produce surface bound H-atoms. It is known that non-defective Al surfaces split only a fraction of surface incoming H_2_(g) with a considerable energy barrier leading to the formation of low coverages of surface bound H-atoms^12–14^. However, clusters and other surface defects such as adatoms can readily dissociate H_2_(g) with considerably lower energy barriers and drive the formation of higher coverages of surface bound H-atoms^15–17^. This has implications in the properties of the interfaces between Al and its environment, and also in the bulk material because Al is susceptible to hydrogen embrittlement^18^. Hence, exposure of Al to H_2_(g) or to atomic hydrogen, can generate surface bound H-atoms that can subsequently diffuse into the bulk of the material. In this context, understanding the interactions between H-atoms and polycrystalline fcc Al with its commonly occurring extended defects such as grain boundaries (GBs) is of utmost importance.

The mechanisms of hydrogen embrittlement of Al are those typically found for other metals that are subject to the same detrimental process^18^. The H-atoms in the lattice of Al can form hydrides^15^, can interact with vacancies^15,19–21^ leading to the stabilization of these in the material and can also accumulate at other defects leading to changes in local plasticity^18,22^. Ultimately the strong interactions between H-atoms and vacancies can lead to the formation of pores both during manufacturing and operation^23–27^. Because vacancies are necessary for the absorption^28^ and diffusion^29,30^ of substitutional impurities, the stabilization that H-atoms impart to vacancies will also have effects on the concentration and distribution of other impurities in the material. Besides the strong interactions with vacancies, H-atoms interact also strongly with dislocations in Al and can accumulate at other lattice defects such as GBs^21,31,32^. An increased concentration of H-atoms at GBs can have severe detrimental effects in the physical–chemical properties of the material, most importantly in the decohesion of grains for example, which is a serious problem if the material is to be used in high pressure vessels^33–35^.

The details of the interactions between H-atoms with Al GBs have not yet been extensively studied^36^. In previous studies we have demonstrated that certain symmetric tilt high-angle low-index GBs of Cu such as the Σ9, Σ5 and Σ11 absorb H-atoms and that the Σ9 and Σ5 GBs can be favorable diffusion channels for these, leading to very fast diffusion^37^. In Al it is known that some GBs have the potential to act as an effective solvent medium for H-atoms^36^. In order to understand the relevance of the interactions between H-atoms and the Al to be used in practical applications it is relevant to employ models of the relevant GBs. Because it is unfeasible to simulate from first principles all GBs that occur in the material, the focus should be on GBs that are frequent and also have structural similarities with other GBs, and even with other defects such as cavities, providing a large excess volume when compared to the bulk. Similarly to fcc Cu also the Σ9, Σ5 and Σ11 GBs are relevant in the microstructure of fcc Al^38^. The Σ3, Σ9 and Σ11 due to their high frequency of occurrence and the Σ5 because of its structure that can resemble other defects^38,39^. We previously found that for Cu however, the Σ3 twin GB cannot trigger the segregation of H-atoms despite its high frequency of occurrence^37^. Hence this GB is not relevant for the mechanisms of hydrogen absorption for Cu, but at present there are no detailed studies on the interactions between H-atoms and these GBs in Al.

Here we found that H-atoms have high preference for sites at the Σ9, Σ5 and Σ11 GBs of Al with larger absorption energies than reported for other metals. Contrary to Cu, the Σ3 GB of Al has the ability to absorb H-atoms even if in limited amounts. This is due to a lower selectivity in bonding between H-atoms in Al as compared to Cu and other metals. These factors are at the origin of the increased concentration of H-atoms at these defects in Al and are the origin of the detrimental physical–chemical–mechanical effects that follow.

## Computational details

### Electronic structure calculations

Density functional theory (DFT) calculations of hydrogen absorption in the bulk and hydrogen segregation at the different GB sites were done with the Vienna ab initio simulation package (VASP 5.4.4)^40^ using the Perdew-Burke-Ernzerhof^41,42^ (PBE) exchange–correlation functional with pseudopotentials consistent with the projector augmented wave^43,44^ (PAW) type and Methfessel-Paxton smearing of order 1 with a width of 0.05 eV. The PBE functional can describe the structure of high-angle low-index symmetric tilt GBs of diverse metals including Al with good accuracy, including also the interactions between these defects and hydrogen^37,45–48^. For all calculations, a plane wave cutoff of 700 eV was employed. Vibrational frequencies were calculated by numerical differentiation of the forces using second-order finite differences with a step size of 0.015 Å. The Hessian matrix was mass-weighted and diagonalized to yield the frequencies and normal modes of the system. For each supercell, the k-point meshes in the Monkhorst–Pack sampling scheme were chosen with basis on the geometry of each supercell in order to produce minimal errors with simultaneous computational efficiency: Σ5 = (6 × 8 × 3); Σ9 = (6 × 8 × 3); Σ11 = (6 × 6 × 3) and Σ3 = (6 × 6 × 3)^49^. The static DFT energies (simply named energies from now onwards) herein reported are electronic energies at 0 K which allow accurate comparisons between bonds and binding energies at sites with similar chemical environment in the solid^50–52^. The segregation energies of the H-atoms (Δ*E*_*n*H_GB_) at the GBs were calculated relative to the bulk as

1 $$\Delta E_{{n{\text{H}}\_{\text{GB}}}} = E_{{n{\text{H}}\_{\text{GB}}}} {-}E_{{n{\text{H}}\_{\text{bulk}}}}$$

where *E*_*n*H_GB_ is the energy of the supercell with *n* H-atoms absorbed to the GB, *E*_*n*H_bulk_ is the energy of *n* H-atoms absorbed to the most stable bulk interstitial site, and Δ*E*_H_GB_ is the corresponding segregation energy per H-atom. Equation (1) considers the dilute limit for *E*_*n*H_bulk_, which implies that this energetic term does not include interactions nor geometrical effects due to neighboring H-atoms at the bulk. This was chosen in order to better mimic the real material where in the bulk non-defective lattice the probability of accumulation of H-atoms at neighboring sites is low for conditions of low concentrations of H in the material. For each GB, the bulk sites are the sites furthest from the GB planes where the absorption energy of the H-atom has converged with respect to the value obtained for a supercell of fcc Al with (3 × 3 × 3) symmetry and 108 atoms. The GB energies (γ_GB_) are defined as

2 $$\gamma_{{{\text{GB}}}} = \, \left( {E_{{{\text{GB}}}} {-}E_{{{\text{bulk}}}} } \right)/{2}A$$

where *E*_GB_ is the energy of the supercell that models the GB, *E*_bulk_ is the total energy of a supercell of Al single crystal that contains a similar number of atoms as those present in the supercell used for modelling the GB and *A* is the area of the GB plane in the supercell. The quantity is divided by two to account for the fact that there are two identical GBs in the supercell.

### Ab initio molecular dynamics simulations (AIMD)

Ab initio molecular dynamics simulations (AIMD) were performed on selected structures corresponding to the segregation of a single H-atom per GB: one segregation site for Σ3 and one for Σ9, and the bulk H-atom absorption for both structures that were taken as the reference states similarly to the static DFT cases. These simulations were performed to understand the role of vibrations due to finite temperature in the driving force for hydrogen absorption and segregation, because it is known that when compared to the perfect lattice, some defects in Al give rise to new vibrational modes characterized by large momenta^53^.

The stable structures for these segregation modes of single H-atoms at Σ3 and Σ9 were simulated at 298 K using the canonical (*NVT*) ensemble with VASP using the PBE functional and the same models and electronic structure parameters described in Section “Electronic structure calculations”. For the dynamics part, the temperature was controlled with the Nosé-Hoover thermostat^54,55^, with an effective mass corresponding to a Nosé frequency of 6000 cm^−1^, and a time-step of 0.5 fs which is sufficient to describe the vibrational motion of H-atoms with good accuracy^56–58^. In order to minimize the error introduced in the dynamics by nuclear quantum effects related with hydrogen, the mass of hydrogen was replaced by that of deuterium. The systems were first well equilibrated in simulations of around 1.5 ps after which the statistics were collected for at least 1 ps of simulation time.

In these conditions, the AIMD free energy is equivalent to the Helmholtz free energy (*F*) of an ensemble and obtained as

3 $$F=-{k}_{b}T\mathrm{ln}Q$$

where *Q* is the partition function defined as a phase space integral of all spatial and momentum coordinates. For two systems that are similar enough, the free energy of a process or reaction ($\overline{{F }_{r}}$)—which is the difference between the free energies of a product configuration (*F*_*p*_) and that of a reagent or initial configuration (*F*_*r*_)—is given by

4 $$\left({\overline{F} }_{r}\right)=\langle {F}_{p}\rangle -\langle {F}_{r}\rangle ={k}_{B}\mathrm{ln}\left(\frac{{\langle {e}^{E{/k}_{B}T}\rangle }_{p}}{{\langle {e}^{E{/k}_{B}T}\rangle }_{r}}\right)$$

In the case of similar systems the ratio of the expectation values in Eq. (4) is close to unity and the errors of the ensemble averages cancel to a large extent leaving only a residual error. In this framework, the approximations for the determination of *F* used in VASP cancel largely for *F*_*p*_ and *F*_*r*_ and the reported AIMD free energies correspond to the VASP free energies obtained in the simulations. The AIMD free energies (∆$\overline{F }$_*H_GB*_) of segregation were determined with Eq. (1) but where instead of the electronic energies *E* the average free energies ($\overline{F }$) are employed. The H-atom absorption configuration taken as the bulk reference was stable at the tetrahedral site during the simulations.

### Structural analysis

To understand the effects of the local geometry and to rationalize the data for Δ*E*_H_GB_ in terms of geometrical properties of the corresponding GB sites we carried out structural analyses. Spatial tessellation methods are valuable tools for analysis of low symmetry structures where the analytic determination of space groups is non-trivial^59,60^. We recently reported the application of the Voronoi tessellation method for structural analyses of this kind for GBs in Cu^61^, other authors have previously demonstrated the usefulness of tessellation methods for similar studies^46,60^. Because of the size difference between the solvent Al-atom and the solute H-atom, Laguerre–Voronoi diagrams were obtained taking into account the differences in the atomic radii. The computed electronic density of states of a single H-atom at the octahedral and tetrahedral sites shows large bonding peaks at the bottom of the valence band, that overlap with the *s* states of Al, which indicates considerable screening of the H-atom^62,63^ but without the ionic bond type of features of a hydride, nor induced hybridization states in the Al atoms at higher energies—at those energies the hydrogen contribution to the resultant density of electronic states is only residual. This is expected in the dilute limit of H-atoms in the lattice where the screening can occur without the formation of hydrides as traditionally defined^64^. Hence in the Laguerre-Voronoi tessellation the crystallographic van der Waals radii of both Al and H were employed, 2.34 Å and 1.26 Å respectively^65^. Laguerre–Voronoi tessellations of the supercells were performed and the resulting polyhedra were used to determine atomic site dependent quantities such as the volume expansion (*V*_x_) and coordination number (*C*_n_). The volume expansion (*V*_x_, %) of a certain GB site is here defined as relative to the volume of a bulk tetrahedral interstitial site, while *C*_n_ is equal to the number of faces of each Laguerre–Voronoi polyhedron. Histograms showing the *V*_x_ of the different GB sites with respect to the volume of the tetrahedral bulk site are given as Supplementary Information.

The Laguerre–Voronoi tessellation applied to an arrangement of particles partitions space using a set of circles centered at the particles. The intersections between these circles are used to generate cells with the shape of polyhedra. These polyhedra define the domain of the particle in the arrangement considering the particles radii—cell or domain of a given particle consists of all the points for which the power distance to the particle is smaller than the power distance to other particles. Similarly to the Voronoi tessellation, the number of faces of each polyhedron that encapsulates a given atom corresponds to the number of neighbors of that atom. A quantification of symmetry for comparative purposes can be done based on the underlying geometrical principles of the Laguerre-Voronoi tessellation. The higher the symmetry of the geometrical arrangement of the neighbors that surround an atom, the more similar are the areas of the faces that constitute the Laguerre-Voronoi polyhedron that encapsulates that atom, and the closer to a sphere is the polyhedron. From this principle a unitless symmetry quantifying parameter σ_*A*_ which is a measure of the sphericity of a polyhedron that surrounds an atomic site can be defined as

5 $${\upsigma }_{A}=\frac{\sum_{i}\left({Av}_{i}\right)}{{A}_{WS}}$$

where *Av*_*i*_ is the area of *i*th face that constitutes the Laguerre–Voronoi polyhedron that surrounds a given atom and *A*_*WS*_ is the area of the Wigner–Seitz sphere centered at the corresponding atom. Because the Wigner–Seitz sphere and the Wigner–Seitz cell have the same volume^66^, *A*_*WS*_ is easily determined from of the volume of the Laguerre-Voronoi polyhedron which encapsulates the atomic site. The quantity σ_*A*_ as here defined reflects the deviation of the shape of a Laguerre-Voronoi polyhedron from that of a sphere: the smaller is σ_*A*_ for an atomic site, the higher is the symmetry of that site in terms of coordination with nearest neighboring atoms. To quantify the differences in symmetry between a GB segregation site and the bulk interstitial site, σ can be re-defined as a symmetry change relative to the bulk as

6 $$\upsigma =\left({\left(\frac{\sum_{i}\left({Av}_{i}\right)}{{A}_{WS}} \right)}_{GB}-{\left(\frac{\sum_{i}\left({Av}_{i}\right)}{{A}_{WS}}\right)}_{bulk}\right)\times 100$$

where the left side term accounts for the symmetry of the GB site and the term to the right accounts for the symmetry of the bulk site. From the definition in Eq. (4), the larger the σ for a given site the lower is the symmetry of that site when compared to the bulk site symmetry. A negative value of σ means that the corresponding site has higher symmetry than the bulk interstitial site. The values of σ, are given as % to facilitate their reading. The reference bulk site from which σ have been determined is the tetrahedral site.

### Symmetric tilt GB models: Σ9, Σ5, Σ11 and Σ3

**Figure 1:**
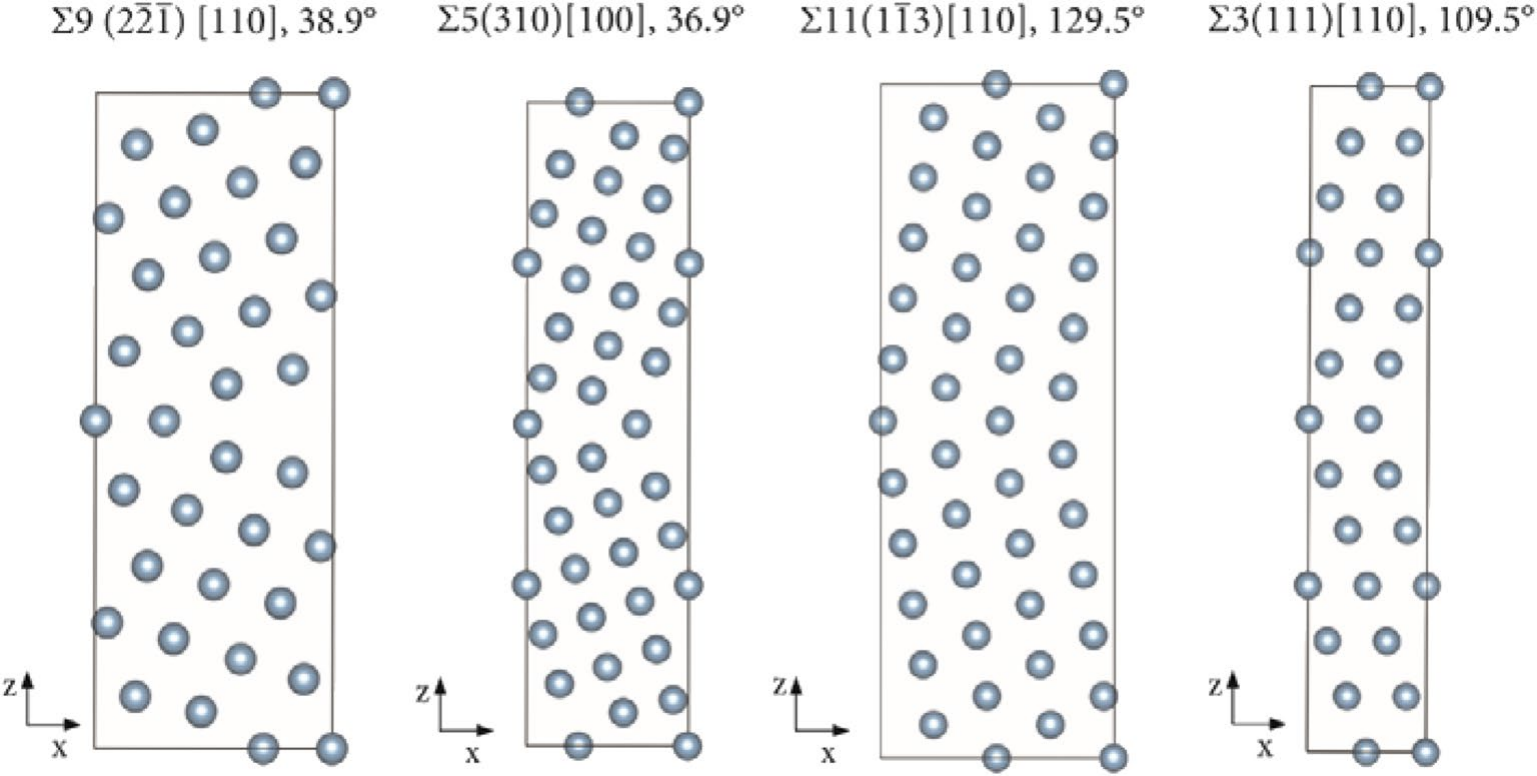
Supercells delimited by black lines showing the geometry optimized Σ9, Σ5, Σ11 and Σ3 GB models, respectively.

The GB models employed in this work are based on models previously employed for the study of the absorption of hydrogen and other impurities in Cu and have been thoroughly benchmarked and tested^37,47^. The same principles were here employed in the creation of the models for Al, taking into account that Al is a p-block element which implies certain modifications within modelling parameters related with the electronic structure. The GB models studied: Σ9(2$\overline{2 }\overline{1 }$)[110], 38.9°; Σ5(310)[100], 36.9°; Σ11(1$\overline{1 }$3)[110], 129.5° and Σ3(111)[110], 109.5°, were built using the coincidence site lattice (CSL) approach from fcc Al with a lattice parameter of 4.04958 Å^67^. The approach is efficient for the construction of symmetric low index CSL GBs consisting of periodic structures of single crystals that have a high density of coincidence sites in the grain boundary plane^68,69^. Each GB was modelled with periodic supercells each containing two oppositely oriented symmetric tilt GBs. After the models were created a geometry optimization was performed. This step consisted of optimizing the supercell size along the normal to the GB planes followed by an optimization of the internal coordinates of the atoms. These two types of optimization were iterated until convergence was reached. All atoms were allowed to relax during these optimizations. For the study of the absorption of H-atoms all atoms were allowed to relax and the geometry optimizations consisted of the optimization of the internal coordinates of the atoms. The geometries have been considered optimized when the self-consistent field (SCF) electronic energy change was smaller than 1 × 10^–5^ eV between cycles and the force acting on each of the atoms smaller than 0.002 eV Å^−1^. The geometry optimized GB models here employed showing examples of absorbed H-atoms are shown in Fig. 1 and their properties are summarized in Table 1.

**Table 1:** Properties of the GBs Σ9(2$\overline{2 }\overline{1 }$)[110], 38.9°; Σ5(310)[100], 36.9°; Σ11(1$\overline{1 }$3)[110], 129.5 and Σ3(111)[110], 109.5°.

GB model	GB area (x, y plane) (Å^2^)	GB energy mJ m^−2^	Atoms in the supercell	Excess volume of the GB Å^3^/Å^2^
Σ9	55.05	430	68	0.120
Σ5	94.35	282	76	0.227
Σ11	53.87	147	88	0.099
Σ3	28.32	10	48	0.046

The obtained GB energies shown in Table 1 are in excellent agreement with previously published literature data obtained from DFT computations and experimental measurements^38,39,70–74^. The GB models of Fig. 1 have shown the necessary stability for the study of high concentrations of H-atoms at the GB planes and in their vicinity. The Σ3 model is smaller than the other GB models due to the higher symmetry of this GB when compared to the others here studied. Overall, the fact that H-atoms are small in comparison with the Al-atoms of the solvent matrix is important to impart stability to the models for the study of high concentrations of hydrogen. This is true despite the considerable local restructuring that the H-atoms cause at certain absorption sites because the H-atoms do not induce large global reconstructions of the GB models. This is not possible with solute atoms or higher concentrations that induce considerable global reconstructions of the supercells. In such cases larger models of the solvent matrix are necessary as previously discussed^46,61^.

## Results and discussion

### Absorption of H at the bulk fcc Al

We started by scrutinizing the most favorable absorption sites for a single H-atom at the bulk fcc Al. While for many single component metallic matrices there are no longer doubts regarding the preferred lattice site for absorption of H-atoms in the single crystal, for fcc Al there are fairly recent accounts that report different preferences for H-atom absorption between the tetrahedral and octahedral sites^75,76^. This is because the difference in absorption energy between both sites is very small for Al when compared with other metals. In this study we obtained Δ*E*_H_tet_ = − 1.730 eV and Δ*E*_H_oct_ = − 1.623 eV for absorption of a single H-atom at the tetrahedral and octahedral sites respectively with makes for a difference of only − 0.107 eV. Note that here we introduce “absorption energy”, defined relative to atomic hydrogen, to distinguish it from “solution energy” defined relative to molecular hydrogen. The addition of the zero-point vibrational energy leads to the same absorption enthalpy—difference of 0.000 eV—for both interstitial sites in Al. The values for the segregation of H-atoms at the GBs have been determined with respect to the bulk tetrahedral site because here we are considering the electronic energies and from this perspective this is the preferred absorption site according to recent literature^32,77^.

### Segregation of single H-atoms to the Σ9, Σ5, Σ11 and Σ3 GBs

**Figure 2:**
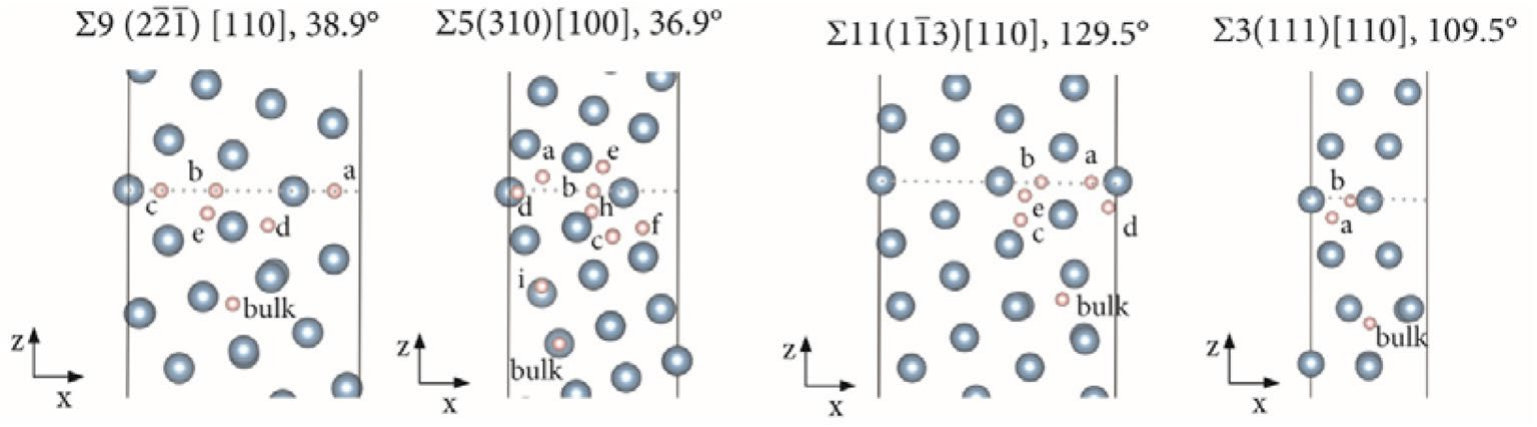
Zoomed in supercells at the GB centra showing the geometry optimized segregation sites for single H-atoms. Each H-atom corresponds to a unique supercell structure, all sites are shown in the same pictures for simplification. Al (filled grey circles), H (filled white circles). The dashed lines highlight the GB centra.

The study of the segregation of H-atoms at the GBs with respect to the bulk tetrahedral site started with the determination of the Δ*E*_H_GB_ for single atoms—one H-atom at the time—at all possible octahedral and tetrahedral like sites of the GBs. For this, a single H-atom was placed at the different binding sites of the GBs, from the GB plane up to the bulk interstitial site and Δ*E*_H_GB_ were determined. Only the favorable segregation sites are here discussed and shown. The favorable segregation sites for single H-atoms are shown in Fig. 2.

**Figure 3:**
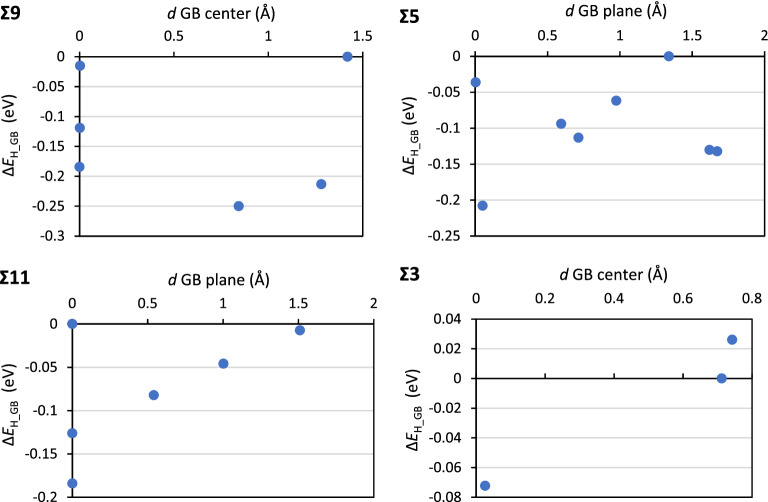
Segregation energies (Δ*E*_H_GB_) as a function of the distance of the corresponding segregation sites from the GB plane (*d* GB plane).

Hydrogen exhibits a clear preference for binding at the GB plane or in the near vicinity. As previously reported by other authors^36^, zero values for Δ*E*_H_GB_ are reached at distances very close to the GB planes as shown by the H-atoms highlighted by “bulk” in the figures. Single H-atoms are capable of driving some reconstruction of the geometry of the nearest neighboring Al-atoms, a phenomenon that we did not observe for these GBs in Cu^37^ but that has been reported for other metals^78^. Another important difference between Al and Cu is that while the Σ3 in Cu does not segregate H-atoms, it can segregate H-atoms in Al. The data shown in Fig. 3 represents the Δ*E*_H_GB_ as a function of distance from the GB plane for each GB.

**Figure 4:**
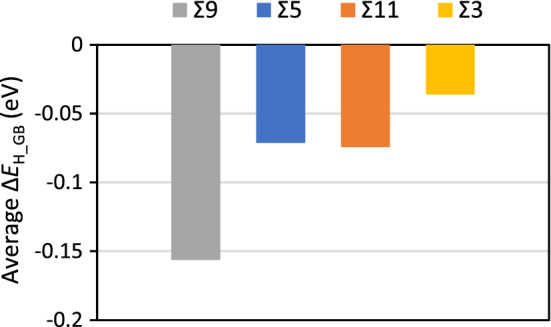
Average segregation energies (Δ*E*_H_GB_) for the different GB studied.

Our obtained Δ*E*_H_GB_ are in excellent agreement with previously reported data^36^. For all GBs with exception of Σ9, the most stable binding sites for H are at the GB plane. Δ*E*_H_GB_ decay very fast towards zero—no driving force for segregation—with distance from the GB plane. The average Δ*E*_H_GB_ of Fig. 4 show that the Σ9 has the largest ability for stabilizing H-atoms with a considerable difference between this GB and the others. The Σ5 and Σ11 have similar average Δ*E*_H_GB_ for single atoms, while the Σ3 GB model has one site capable of attracting H-atoms from the bulk, but with a very small Δ*E*_H_GB_. Due to the symmetry of the Σ3 GB model this corresponds to 50% of the sites.

In order to understand if such small segregation energies can still stabilize the H-atoms and drive their segregation at room temperature we performed AIMD simulations in selected structures. We chose one structure that has a Δ*E*_H_GB_ very close to zero and on that has a segregation site with a symmetry that allows for easy outwards diffusion of H from the site and simulated the systems at room temperature. The structures chosen were those where the H-atoms are bound at the site *e* of Σ9 and site *b* of Σ3. The AIMD segregation free energies Δ$\overline{F }$_H_GB_ and the corresponding data obtained with DFT for comparison are shown in Table 2.

**Table 2:** Segregation energies (Δ*E*_H_GB_) obtained with static DFT at 0 K and average segregation free energies (Δ$\overline{F }$_H_GB_) obtained with AIMD at *T* = 298 K for absorption of single H-atoms to site e of Σ9 and site b of Σ3.

GB	H-segregation site	Static DFT Δ*E*_H_GB_ (eV)	AIMD Δ$\overline{{\varvec{F}} }$_H_GB_ (eV)
Σ9	*e*	− 0.250	− 0.136
Σ3	*b*	− 0.072	0.079

The addition of temperature-induced dynamical motion leads to a decrease of the driving force for segregation. This was expected because of the known appearance of vibrational modes in Al GBs that besides reaching high frequencies and carrying significant momenta, also contain a certain degree of anharmonicity^53^. The combination of these two factors leads to a decrease in the driving force for segregation of H-atoms. The resulting effect is that while segregation is predicted by static DFT to be favorable at the site *b* of Σ3, it is not favorable when the vibrational motion at 298 K is considered. However, diffusion of the H-atom away from the site *b* did not occur which indicates that there is a significant barrier for diffusion from that site. This activation energy barrier contributes also to the accumulation of H-atoms at the GBs. Due to the small size of the Σ3 GB model here employed certain long wavelength vibrational modes are not accounted for and those modes could eventually stabilize the segregation of H-atoms at this GB. Because of the very small differences in energies for segregation at Σ3 between static DFT and AIMD data we consider that this GB has a weak driving force for segregation of H-atoms. This contrasts with the Σ9 GB that even in the room temperature AIMD simulations can attract the H-atom to site *e* with a considerable energetic gain with respect to a bulk interstitial site.

### Segregation of multiple H-atoms to the Σ9, Σ5, Σ11 and Σ3 GBs

**Figure 5:**
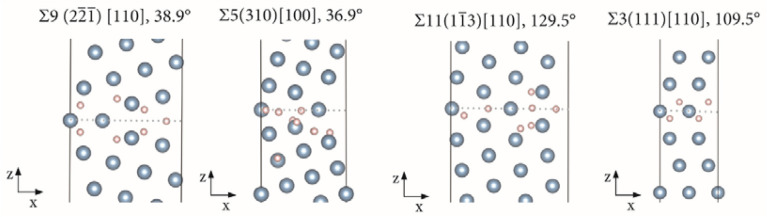
Supercells zoomed in at the GB centra showing the geometry optimized structures with the maximum number of H-atoms that the GBs can absorb. The increase of the number of H-atoms at the GBs started at the most stable sites which typically are at the GB planes and proceeded towards the bulk as the number of H-atoms was increased. Al (filled grey circles), H (filled white circles). The dashed lines highlight the GB centra.

The hydrogen sorption capacity of the GBs was studied by increasing the concentration of H—sequentially filling the most stable binding sites found for single H-atoms, adding subsequently H-atoms at each stage to the most stable configurations for 2, 3, 4 etc. H-atoms—until the GBs were completely filled reaching the maximum absorption capacity of H-atoms with respect to the bulk absorption energy. The GB models fully saturated with H-atoms are shown in Fig. 5.

**Figure 6:**
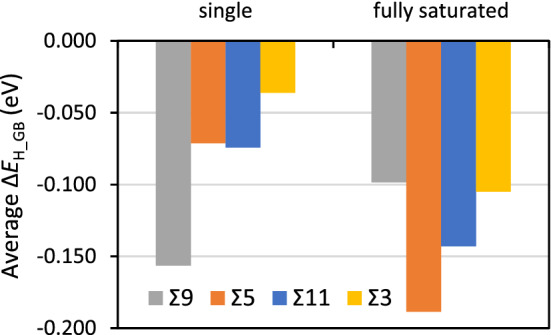
Average segregation energies per H-atom (Δ*E*_H_GB_) for segregation of single H-atoms (one H-atom at the time) and for the GBs fully saturated with H-atoms.

A comparison between the structures for the maximum number of H-atoms (Fig. 5) and those obtained for single H-atoms (Fig. 2) shows that with increasing number of H-atoms, absorption sites that were initially preferred for single H-atom are no longer preferred when more H-atoms are in the vicinity, and vice versa. This is due to neighboring or cooperative effects and has been previously observed for H at Cu GBs and H and other atoms at Al surfaces^37,79,80^. These cooperative neighboring effects are known to occur for some adsorbates at surfaces and depending on geometric and electronic structure effects can lead to either weaker or stronger adsorption with increasing coverage^81^. For these GBs, the occurrence of these effects is seen in Fig. 6 where the shifts in average Δ*E*_H_GB_/H-atom for segregation of one H-atom at the time—single H-atoms—and for the GB models fully saturated with H-atoms are shown. For all GBs segregation of multiple H-atoms leading to a fully saturated GB caused the average value of Δ*E*_H_GB_/H-atom to become more negative which implies a stronger driving force for segregation. Σ9 is the exception to this and the neighboring effects in absorption lead to a smaller driving force for segregation, however the resulting Δ*E*_H_GB_/H-atom for the fully saturated GB is still considerable and reaches − 0.100 eV. In Σ9, the GB center has sites with large (*V*_*x*_) % and smaller *C*_*n*_ than Σ5 for example. These two effects combined make incoming H-atoms be able to displace the H-atoms bound at the GB center shown in Fig. 2, leading to a symmetrical disposition of H-atoms for Σ9, a situation that does not occur for the other GBs. Additionally, DFT predicts that Σ3 does not have a significant ability to drive the segregation of single H-atoms but when the GB is completely filled with H, the average Δ*E*_H_GB_ become three times larger: -0.105 eV/H-atom. These effects are further highlighted by the data on the incremental segregation energies of the H-atoms given in the Supplementary Information.

Overall, the large differences between the structures of Figs. 2 and 5 and between the energetic data of Fig. 6 for fully saturated GBs and those obtained for single H-atoms absorption show that the extrapolation of segregation data for single atom to higher concentrations ignoring neighboring effects should not be done because it is accompanied by considerable errors. The maximum number of H-atoms that each GB model can accommodate per area of GB are given in Table 3.

**Table 3:** GB plane area (Å^2^) per one H atom for the fully saturated segregation at each GB.

GB type	Σ9	Σ5	Σ11	Σ3
GB plane area (Å^2^) per 1 H atom	Al			
	3.51	2.02	2.96	7.08
	Cu			
	n/a	13.93	20.29	No segregation

Data for Cu for comparison, retrieved from^37^. Both sets of data consider segregation at both sides of the GB plane.

Contrary to Cu, these GBs in Al can accommodate considerably higher concentrations of H-atoms per unit area of GB plane. Σ3 in Cu does not drive the uptake of H-atoms, and data for Σ9 is not available. But the fact that all GBs of Al can uptake significant amounts of H-atoms can partly explain the embrittlement of Al, while for Cu no hydrogen embrittlement of clean GBs is known to happen^37^. The data also shows that all the GBs in Al have very similar maximum concentrations of H-atoms, which will lead to a close to constant distribution of H-atoms in the GBs of the material which in turn can have detrimental effects in the mechanical properties. However it is also necessary to consider the magnitude of the Δ*E*_H_GB_/H-atom and in this case the values for Al are considerably larger than for Cu^37^ but similar to the data obtained for Ni^82^. These results also suggest that it is difficult to control the uptake of hydrogen by Al using for this purpose the control of the distribution of specific GBs in the material—processing to create specific distributions of certain types of GBs, as for example GB engineering^83^. This is because due to both their frequency of occurrence and geometry, the GBs here studied can represent a large number of different types of such defects in Al.

### Structural effects in the uptake of H-atoms by the GBs

**Figure 7:**
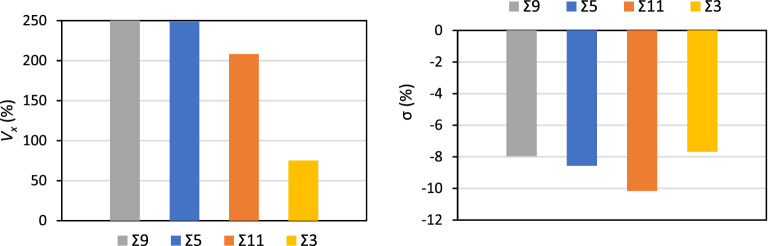
Average volume expansion (*V*_*x*_) % and symmetry quantifying parameter σ (%) of the interstitial atomic sites at the GBs with respect to the bulk.

The local geometry of the GBs is decisive for the segregation of impurities^37,46,47^. The descriptors derived from the Laguerre-Voronoi tessellation described in the Section “Structural analysis” are powerful mathematical tools for the rationalization of complex geometries in terms of their effects on chemical bonding. The average *V*_*x*_ and σ for the interstitial sites that have the ability to accommodate segregating H-atoms at each GB are shown in Fig. 7. The average *V*_*x*_ are similar for the Σ9, Σ5 and Σ11 and considerably smaller for the Σ3. We recall that the Laguerre-Voronoi tessellation method considers the different atomic radii of Al and H, with H occupying the interstitial lattice sites. This can lead to large *V*_*x*_ as a result of certain geometrical changes, because in this case the values are more sensitive to those changes than for the case of the Voronoi tessellation for a system composed solely of particles of one type. The σ show that the average geometry of the interstitial atomic sites at the GBs is closer to spherical than that of the bulk tetrahedral site. The fact that H-atoms tend in general to be stabilized by a higher symmetry of the spatial disposition of ligands can explain the tendency of these GBs to stabilize and segregate H-atoms.

**Figure 8:**
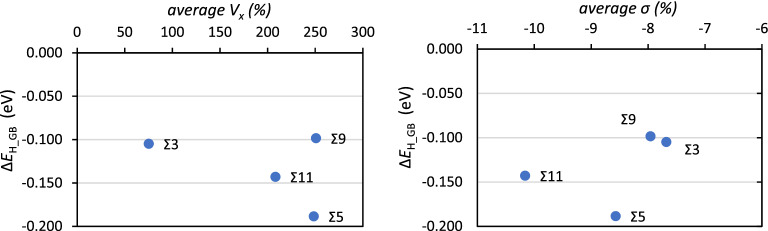
Average segregation energies per H-atom (Δ*E*_H_GB_) as a function of the average *V*_*x*_ (%) and average σ (%) of the interstitial atomic sites for each GB.

**Figure 9:**
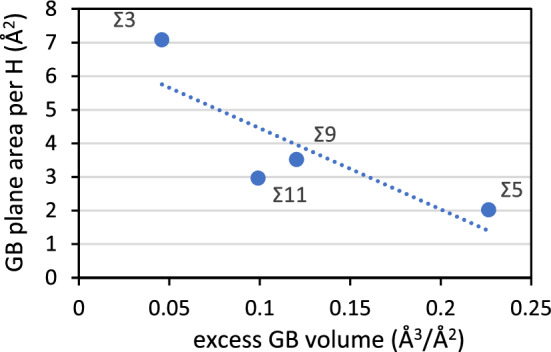
Correlation between the excess GB volume (Å^3^/A^2^) and the inverse concentration of H-atoms at the GBs, expressed as GB plane area per 1 H-atom (Å^2^). The correlation follows: y = − 24.14x + 6.86 with a correlation coefficient of 0.69.

The correlation between the average *V*_*x*_ for each GB and the average Δ*E*_H_GB_ per H-atom for fully saturated GBs are shown in Fig. 8. The data of Fig. 8 shows that there is a fairly linear correlation between Δ*E*_H_GB_ and *V*_*x*_ for Σ3, Σ11 and Σ5 with a larger average volume expansion leading to stronger bonding of the H-atoms for these GBs. In this correlation the Σ9 is the outlier and this can be related with the fact that this GB is also an outlier with regard to the cooperative effects in the uptake of H-atoms as shown in Fig. 6. The σ show a global trend where a lower symmetry—less negative σ leads to weaker bonding with H but overall there is a fairly close to linear correlation between the excess GB volume and the inverse concentration of H-atoms at the GBs, expressed as GB plane area per 1 H-atom (Å^2^), shown in Fig. 9.

**Figure 10:**
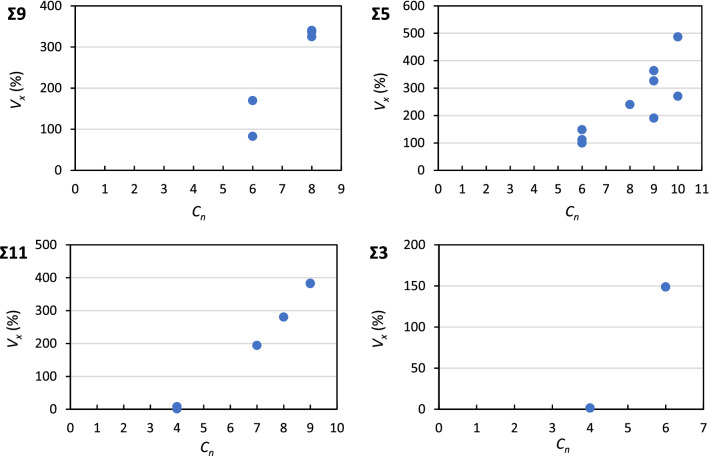
Volume expansion relative to the bulk tetrahedral site (*V*_*x*_, %) as a function of the coordination number (*C*_*n*_) for the GB sites where H-atom segregation is favorable.

It can be seen in Fig. 10 that for these GBs there is a strong correlation between *V*_*x*_ and *C*_*n*_ with a positive correlation coefficient. This implies that the sites with larger coordination number also have a larger volume expansion and the combined effect leads to an increase in the stability of H-atoms relative to the bulk. Additionally, as mentioned above the atomic environment of many segregation sites at the GBs has higher symmetry—more spherical—than at the bulk tetrahedral site, which is largely explained by the larger *V*_*x*_, and *C*_*n*_ associated with those sites. The overall effect is that the local atomic environment at these commonly occurring GBs in Al favors the segregation of H-atoms. Because for all GBs here studied the correlations between *V*_*x*_, and *C*_*n*_ and in turn with Δ*E*_H_GB_ are so strong, this suggests that it is difficult to control the atomic structure of interstitial sites at GBs in Al in order to mitigate the segregation of H-atoms.

## Conclusions

Single H-atoms can cause displacements of Al-atoms at the Σ9, Σ5, Σ11 and Σ3 GBs of Al, a phenomenon not typically found for H in transition metals. For single H-atoms all favorable segregation sites are at the GB planes and the segregation tendency decreases steeply with distance from these. No segregation tendency was found at distances larger than 1.5 Å from the GB plane for Σ9, Σ5, Σ11, while for Σ3 the segregation of single H-atoms is only favorable at the GB plane. However, there are large cooperative effects that lead to an increase in segregation when other H-atoms are bound at neighboring sites. This results in high concentrations of H-atoms at these GBs with considerable segregation energy per atom. All GBs have similar upper concentration limits of H-atoms per area of GB plane which indicates a tendency for uniform distribution of H-atoms at the GBs independently of the type of GB.

The structural analyses based on geometry descriptors derived from the Laguerre–Voronoi tessellation method show that the larger fraction of interstitial atomic sites at these GBs has higher symmetry than a bulk tetrahedral site. Additionally, many of those sites have also large volume expansions and coordination numbers when compared to the bulk site. We found close to linear correlations between volume expansion and coordination numbers at the GBs which combined with the higher symmetry of many sites are at the origin of the increased driving force for H-atom segregation at these GBs of Al when compared to other metals. Overall these results suggest that attempting to mitigate hydrogen uptake by controlling the frequency of occurrence of certain GBs may not be the most efficient method for Al.

## Supplementary Information

Supplementary Information.

## Data Availability

The data generated is available from the corresponding author upon reasonable request.
