# Supplementary material for: Hydrogen at symmetric tilt grain boundaries in aluminum: segregation energies and structural features
Source: Sci Rep. 2022 Nov 18;12:19872. doi: 10.1038/s41598-022-23535-9 (PMC9674852; doi:10.1038/s41598-022-23535-9)
Supplement: Supplementary file 1 — Supplementary Information. [file 41598_2022_23535_MOESM1_ESM.docx]

**Hydrogen at symmetric tilt grain boundaries in aluminum: Segregation energies and structural features**

Cláudio M. Lousada,* and Pavel A. Korzhavyi

Department of Materials Science and Engineering, KTH Royal Institute of Technology, SE-100 44 Stockholm, Sweden

* Correspondence: cmlp@kth.se;

**Supplementary information**

**S1.1 Excess volume at the GB sites**

Figure 1. Histograms showing the excess volume *V_x_* (%) of the different GB sites with respect to the volume of the tetrahedral bulk site (0%).

**S1.2. Incremental segregation energies of H-atoms**

The incremental segregation energies of the H-atoms (Δ*E_n_*_H_GB_inc_) have been determined as

Δ*E_n_*_H_GB_inc_ = (*E_n_*_H_GB_ – *E_(n-1)_*_H_GB_) – (*E_n_*_H_bulk_ - *E*_bulk_) (S1)

where *E_n_*_H_GB_ is the energy of the supercell with n H-atoms at the GB, *E_(n-1)_*_H_GB_ is the energy of the most stable configuration of H-atoms in the supercell corresponding to *n-1* H-atoms at the GB, *E_n_*_H_bulk_ is the energy of the most stable configuration of H-atoms in the supercell corresponding to *n* H-atoms bound to the bulk tetrahedral site and *E*_bulk_ is the energy of the supercell without H-atoms. The Δ*E_n_*_H_GB_inc_ expressed according to eq S1 gives the energy of the addition of an H-atom relative to the previous configuration and is not a measure of the absolute driving force for segregation. This is because even though this quantity is positive for decreasing driving force for segregation, the quantity Δ*E_n_*_H_GB_ is still negative with respect to the bulk for the cases where segregation is possible.

Table S1. Δ*E_n_*_H_GB_ and Δ*E_n_*_H_GB_inc_ for the segregation of increasing numbers (*n*) of H-atoms to the GBs. For Σ5 the limit of 16 atoms is reached as the H-atoms bound further from the GB center are already on sites that have the same Δ*E_n_*_H_GB_ as the bulk for single H-atom segregation.

|  | Σ5 | Σ 5 | Σ 11 | Σ 11 | Σ 9 | Σ 9 | Σ 3 | Σ 3 |
| --- | --- | --- | --- | --- | --- | --- | --- | --- |
| *n* H-atoms | Δ*E_n_*_H_GB_ | Δ*E_n_*_H_GB_inc_ | Δ*E_n_*_H_GB_ | Δ*E_n_*_H_GB_inc_ | Δ*E_n_*_H_GB_ | Δ*E_n_*_H_GB_inc_ | Δ*E_n_*_H_GB_ | Δ*E_n_*_H_GB_inc_ |
| 1 | -0.208 |  | -0.184 |  | -0.250 |  | -0.072 |  |
| 2 | -0.204 | 0.004 | -0.190 | -0.006 | -0.258 | -0.008 | 0.002 | 0.074 |
| 3 | -0.190 | 0.014 | -0.148 | 0.042 | -0.234 | 0.024 | -- | -- |
| 4 | -0.192 | -0.002 | -0.106 | 0.042 | -0.248 | -0.014 | -- | -- |
| 5 | -0.212 | -0.020 | -- | -- | -0.237 | 0.012 | -- | -- |
| 6 | -0.192 | 0.020 | -- | -- | -0.228 | 0.009 | -- | -- |
| 7 | -0.200 | -0.008 | -- | -- | -0.200 | 0.028 | -- | -- |
| 8 | -0.189 | 0.011 | -- | -- | -0.198 | 0.002 | -- | -- |
| 9 | -0.191 | -0.002 | -- | -- | -0.176 | 0.022 | -- | -- |
| 10 | -0.202 | -0.011 | -- | -- | -0.194 | -0.018 | -- | -- |
| 11 | -0.198 | 0.004 | -- | -- | -0.160 | 0.034 | -- | -- |
| 12 | -0.188 | 0.010 | -- | -- | -0.130 | 0.030 | -- | -- |
| 13 | -0.201 | -0.013 | -- | -- | -0.110 | 0.020 | -- | -- |
| 14 | -0.198 | 0.003 | -- | -- | -0.098 | 0.012 | -- | -- |
| 15 | -0.192 | 0.006 | -- | -- | -- | -- | -- | -- |
| 16 | -0.189 | 0.003 | -- | -- | -- | -- | -- | -- |

Figure 2. Δ*E*_H_GB_ per H-atom (eV) as a function of the number of H-atoms segregated to the GBs.

Figure 3. Incremental segregation energies (Δ*E_n_*_H_GB_inc_) (eV) as a function of the number of H-atoms segregated to the GBs.
